# Global Health Security: A Blueprint for the Future

**DOI:** 10.3201/eid2812.221035

**Published:** 2022-12

**Authors:** Katherine M. Bianchi

**Affiliations:** Biomedical Advanced Research & Development Authority, Office of the Assistant Secretary for Preparedness and Response, US Department of Health and Human Services, Washington, DC, USA

**Keywords:** infectious diseases, global health, disease prevention and control, antimicrobial resistance, bioterrorism and preparedness, vector-borne infections, mosquito-borne infections, malaria

## Lawrence O. Gostin

## Global Health Security: A Blueprint for the Future

## Harvard University Press, Cambridge, Massachusetts, USA, 2021;

## ISBN: 9780674976610 (cloth);

## Pages: 352; Price: $45

Lawrence Gostin’s Global Health Security: A Blueprint for the Future comes along at an opportune time, as a pandemic reminds humankind of the importance of public health response to our wellbeing and security (Figure). The book addresses the types of infectious disease outbreaks and actions needed to prepare and respond, emphasizing the roles of multinational agreements and international cooperation. For readers knowledgeable about global health security, the content might serve as a refresher, for persons unfamiliar with the subject, as an introduction. Gostin, director of Georgetown Law School’s O’Neill Institute for National and Global Health Law, shaped by experiences as a lawyer and interactions with the World Health Organization, examines scientific and policy approaches. He discusses COVID-19 throughout the book and emphasizes health equity, drawing attention towards disadvantaged populations in low- and middle-income countries 

**Figure:**
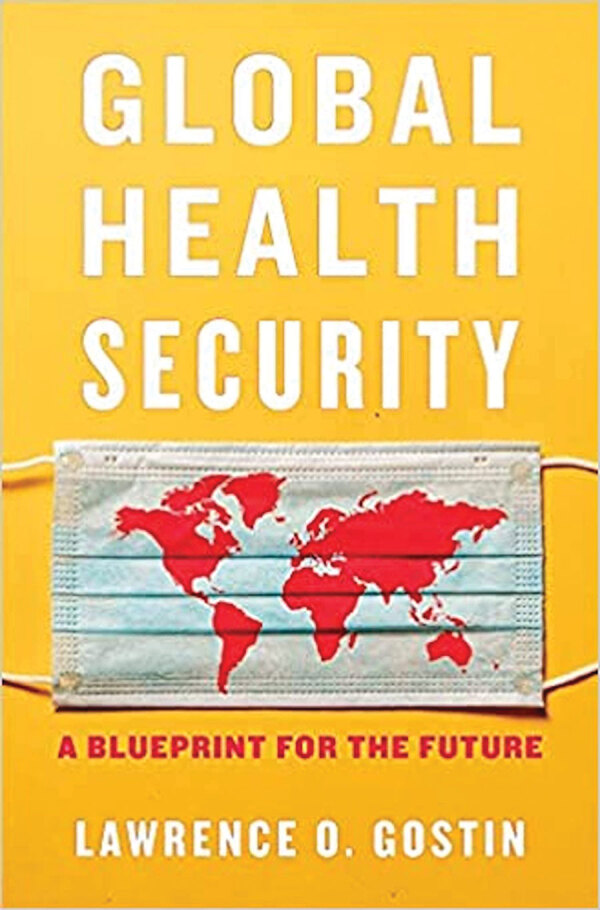
Global Health Security: A Blueprint for the Future

The book is separated into 2 sections: “Growing Threats” encompasses topics as diverse as mosquitoborne diseases, climate change, and biosafety and biosecurity. “From Risk to Action” focuses on global health security governance, pathogen sharing, universal health coverage, and developing and using medical countermeasures. The chapter aptly titled “Humanity’s Biggest Killer” outlines the history, epidemiology, and clinical manifestations of mosquitoborne diseases. Gostin discusses interventions such as genetically engineered mosquitoes, long-lasting insecticidal bed nets, and vaccination, but cautions “optimism should be tempered…by the limits of traditional strategies, hurdles still to overcome for newer possibilities (from scientific challenges to special publics), and the weaknesses of the health systems.” In another chapter he discusses how behavior such as prophylactic use of antimicrobials in livestock and their overprescription by clinicians contributes to antimicrobial resistance and how lack of economic incentives for private sector investment inhibits development of antimicrobials. Gostin drives home potential dangers from complacency: “Imagine if we lived in a world where once fully treatable infections became life-threatening and routine surgeries posed lethal risks.”

The final chapter, “Global Medical War Chest,” focuses on regulatory considerations, market incentives, and clinical trial design for medical countermeasure development. Gostin argues that “financing, law, and ethics must be in place—not just when an outbreak strikes, but more importantly during periods of calm…Collectively, policies and processes that support all the building blocks of research and development can save millions of lives.” 

Gostin might have highlighted certain topics in greater detail, such as misinformation and disinformation and transformative technologies. Additional information about cultural influences on health would have been beneficial, as would perspective on the history of combatting outbreaks, such as through use of face masks. On the other hand, given that global coordination is critical because infectious disease spread does not recognize geographic borders, information about multinational policies and governance is particularly insightful. The author’s use of first-person perspective and storytelling helps keep readers emotionally invested, and the tabletop exercise outbreak scenarios, list of health entity abbreviations, and headings used to guide the reader are valuable additions. 

Global Health Security could be beneficial for policymakers reflecting on lessons learned from the COVID-19 pandemic and considering strategies to combat future outbreaks. Gostin’s perspective provides an important lesson: “When the world fully funds and thoroughly prepares for dangerous outbreaks, it is highly likely that dangerous pathogens can be rapidly brought under control. If we neglect the threat, wait until it is too large to stop, and then panic, many lives, and dollars, will be lost. Most of the human and economic suffering is preventable.” 

